# The effect of assessing genetic risk of prostate cancer on the use of PSA tests in primary care: A cluster randomized controlled trial

**DOI:** 10.1371/journal.pmed.1003033

**Published:** 2020-02-07

**Authors:** Jacob Fredsøe, Jan Koetsenruyter, Peter Vedsted, Pia Kirkegaard, Michael Væth, Adrian Edwards, Torben F. Ørntoft, Karina D. Sørensen, Flemming Bro

**Affiliations:** 1 Department of Clinical Medicine, Aarhus University, Aarhus, Denmark; 2 Department of Molecular Medicine, Aarhus University Hospital, Aarhus, Denmark; 3 Research Unit for General Practice, The Research Centre for Cancer Diagnosis in Primary Care (Cap), Aarhus University, Aarhus, Denmark; 4 Department of Public Health, Randers Regional Hospital, Randers, Denmark; 5 Department of Public Health, Section of Biostatistics, Aarhus University, Aarhus, Denmark; 6 Division of Population Medicine, School of Medicine, Cardiff University, Cardiff, United Kingdom; National Cancer Institute (U.S)., UNITED STATES

## Abstract

**Background:**

Assessing genetic lifetime risk for prostate cancer has been proposed as a means of risk stratification to identify those for whom prostate-specific antigen (PSA) testing is likely to be most valuable. This project aimed to test the effect of introducing a genetic test for lifetime risk of prostate cancer in general practice on future PSA testing.

**Methods and findings:**

We performed a cluster randomized controlled trial with randomization at the level of general practices (73 in each of two arms) in the Central Region (Region Midtjylland) of Denmark. In intervention practices, men were offered a genetic test (based on genotyping of 33 risk-associated single nucleotide polymorphisms) in addition to the standard PSA test that informed them about lifetime genetic risk of prostate cancer and distinguished between “normal” and “high” risk. The primary outcome was the proportion of men having a repeated PSA test within 2 years. A multilevel logistic regression model was used to test the association.

After applying the exclusion criteria, 3,558 men were recruited in intervention practices, with 1,235 (34.7%) receiving the genetic test, and 4,242 men were recruited in control practices. Men with high genetic risk had a higher propensity for repeated PSA testing within 2 years than men with normal genetic risk (odds ratio [OR] = 8.94, *p* < 0.01). The study was conducted in routine practice and had some selection bias, which is evidenced by the relatively large proportion of younger and higher income participants taking the genetic test.

**Conclusions:**

Providing general practitioners (GPs) with access to a genetic test to assess lifetime risk of prostate cancer did not reduce the overall number of future PSA tests. However, among men who had a genetic test, knowledge of genetic risk significantly influenced future PSA testing.

**Trial registration:**

This study is registered with ClinicalTrials.gov, number NCT01739062.

## Abstract 2

Jacob Fredsø and colleagues carried out a cluster randomised trial in Denmark to assess whether having the genetic test to check for prostate cancer risk influenced the number of prostate cancer antigen (PSA) tests in future.

## Author summary

**Why was this study done?:**

- Population-based systematic screening and opportunistic screening for prostate cancer using the prostate-specific antigen (PSA) test result in overdetection and overtreatment of indolent cancers, which would not result in symptoms during the patient’s normal life span.
- Utilizing a genetic prostate cancer risk assessment could help increase future PSA testing in men with high lifetime risk and reduce it in men without normal lifetime risk.

**What did the researchers do and find?:**

- We provided a lifetime risk assessment (either high or normal risk) for prostate cancer in general practice (1,235 patients tested).
- We compared the proportion of patients with high/normal risk who had a repeat PSA test within 2 years with 4,242 patients (controls) who did not have the genetic test.
- As compared with controls, patients with a high genetic risk had a much higher chance of a repeat PSA test within 2 years (8.94 times higher), whereas patients with a normal genetic risk had a lower chance of a repeat PSA test (0.62 times lower).

**What do these findings mean?:**

- By providing a genetic risk assessment for prostate cancer in general practice, it was possible to change the patients’ future use of PSA testing—i.e., targeting PSA testing to the group of men with high risk while reducing opportunistic testing in men at normal risk.
- Longer follow-up is required to evaluate the effect of genetic risk assessment on the number of prostate cancer biopsies and diagnoses.

## Introduction

Prostate cancer is the most common cancer among men in Europe, affecting about 190,000 men for the first time every year and causing about 80,000 deaths [1,2]. A commonly used method for early detection of prostate cancer is the prostate-specific antigen (PSA) test, although this method has limited accuracy [3]. This results in both failure to detect genuinely aggressive cancers and overdetection and overtreatment of indolent prostate cancers. Currently, although still debated, population-based organized screening using the PSA test is not advised [4,5]. Opportunistic screening is increasingly common, with some indications that overdiagnosis is even more profound here than for organized screening [6,7]. Therefore, there is interest in whether PSA testing can be targeted to those for whom it may be most valuable.

Risk stratification is proposed as a strategy to reduce the overall number of men having the PSA test and to improve the benefit-to-harm ratio by targeting those most likely to benefit [8]. This is already advocated in guidelines assessing risk by family history [4,9]. Now, genetic markers offer the potential to improve risk assessment for developing prostate cancer. So far, scientific advances in genome-wide association studies have identified more than 200 genetic variants for higher prostate cancer risk, the so-called single nucleotide polymorphisms (SNPs) [10–13]. It has been estimated that the currently known risk-SNPs together explain approximately 33% of the familial risk of prostate cancer in populations of European ancestry and that the top 10% of men in the risk distribution have a 2.9-fold increased relative risk of prostate cancer compared with the general population [11,14]. Retrospective studies comparing nongenetic risk prediction models for detection of prostate cancer with models containing SNP information showed that the genetic models had higher specificity than the nongenetic models based on PSA level, age, and family history [15,16].

Despite the potential benefits, few studies have used genetic prostate cancer risk assessment in the clinical setting as a tool for improving the use of PSA testing and prostate cancer diagnosis, particularly in general practice, in which most symptoms are presented and testing takes place.

By introducing a genetic lifetime risk test for prostate cancer in general practice, we aimed to investigate its possible effect on future PSA testing, with the hypothesis that the number of PSA tests would increase in the small group of men with “high” risk and could be reduced in the large group of men with “normal” risk [17].

## Methods

### Study design

We performed a cluster randomized controlled trial with randomization at the level of general practices. Block randomization was applied to balance groups with respect to the number of doctors in a practice. The study follows the CONSORT statement (S1 Text) for reporting randomized trials, and the analysis plan along with the study protocol have been described in greater detail previously [17]. All practices knew which arm they were assigned to by design. This study is registered with ClinicalTrials.gov (number NCT01739062).

### Setting and participants

The study took place from February 2013 to November 2014 in Central Region Denmark, which is one of five regions in Denmark and has 1.3 million inhabitants and about 850 general practitioners (GPs) in 400 practices. All Danish residents have free access to GPs, who serve as gatekeepers to the rest of the healthcare system. Over 98% of all citizens are registered with a specific practice. It is mandatory for GPs to use electronic patient records, and test requisitions and test results are transferred electronically [18]. All Danish citizens have a unique personal identifier number, which is registered for all contacts and investigations and which makes it possible to link all data on an individual basis [19].

For both the control and intervention arm, men were eligible for participation if they were aged 18–80 years, registered with one of the randomized practices, had a normal PSA test at inclusion, did not move out of Central Region Denmark, and remained alive at least 2 years after inclusion. A normal PSA test was defined as a value less than 3.0 ng/ml for men below age 60, less than 4.0 ng/ml for men aged 60–70, and less than 5.0 ng/ml for men aged 70 or above. Men were excluded if they had an elevated PSA level at inclusion or within the previous 2 years, known prostate or bladder disease, or a current or previous prostate cancer diagnosis.

In this trial, men of non-European ancestry were included but not offered the genetic test because genetic risk estimates were only based on data from European-descent population studies (S2 Text). For safety reasons, men in the intervention group who had more than one close relative with a prostate cancer diagnosis were advised to undergo systematic screening with PSA tests according to current clinical guidelines in Denmark [20,21]. Both groups, however, were still included in the intention-to-treat analysis. The GP determined eligibility for the genetic test in the clinical setting based on criteria incorporated into the web-based test-ordering system [22].

### Intervention

In addition to a PSA test, men in intervention practices were offered an additional genetic risk assessment. Based on genotyping of 33 risk loci/SNPs (S2 Text), individual lifetime prostate cancer risk was calculated for each man after adjusting for prostate cancer family history [17]. Lifetime risk scores were dichotomized into a normal risk (<30%) and high risk (≥30%) categories, thus conservatively adhering to the Danish recommendations to screen individuals who (based on family history) have an estimated 33% lifetime risk of being diagnosed with prostate cancer [20,21,23,24].

The procedure was as follows: when a man was to have a PSA test and met the inclusion criteria, the GP informed him about the study and provided written information. If he consented to participate, the GP drew an additional 4-ml EDTA-stabilized blood sample, which was sent to the laboratory together with an online request for a genetic risk assessment that included information about the patient’s age and self-reported family history of prostate cancer. Specifically, the patient answered the following question: “Has your father, brother, father’s brother, or mother’s brother been diagnosed with prostate cancer?” The answer options were “None,” “Yes, 1 relative,” “Yes, 2 or more relatives,” or “Unknown.” If the patient’s family disposition was unknown but the total risk assessment, in theory, would change from normal to high in the case of a known positive family history, we reported the total risk assessment result as unknown. Within 2–6 weeks, the laboratory analyzed the genetic test and sent the result electronically to the intervention practice GP with one of the following recommendations:

High lifetime risk:

“The patient belongs to a group with increased risk of developing prostate cancer in the future. If the patient develops prostate cancer in the future, in most cases, the cancer will be slow growing. For early detection, the patient is encouraged to have a yearly PSA test.”

Normal lifetime risk:

“The patient belongs to a group of patients at normal lifetime risk of getting a prostate cancer diagnosis. It is not considered necessary or beneficial for the patient to have more PSA tests in the future, unless the patient develops urinary tract symptoms or one or more of his relatives develops prostate cancer.”

Unknown lifetime risk:

“The risk for developing prostate cancer in the future cannot be estimated due to missing information for family history.”

Once the practice received the genetic risk assessment results (either normal, high, or unknown), the GP or their staff informed the patient about the result by telephone, email, letter, or during consultation. If there were questions, both intervention practice personnel and men could contact a project telephone hotline to reach the researchers. Each intervention practice also received written information with recommendations about follow-up PSA testing according to current guidelines [21] and information about the benefits and shortcomings of genetic risk assessment as a tool to support decision-making about PSA testing for prostate cancer. Patients with an unknown genetic risk were not given any further recommendations, and their future course was at the discretion of their GP.

The GPs were remunerated with an additional 142 DKK (approximately €19) per consultation, which covered the additional work in relation to information and blood sampling. Control practices performed care as usual and received no further information or compensation.

### Outcomes

The primary outcome was the proportion of men who had a PSA test within 2 years after a normal PSA test result at inclusion. We chose 2 years of follow-up because this was considered a reasonable screening interval for healthy adults [25]. Although men were included only through GP practices, tests requested by hospitals and private specialist clinics were also taken into account as a PSA test in the follow-up period.

Secondary outcome was the proportion of men with an elevated PSA test. Data about the date and level of the PSA test were collected from the regional clinical laboratory information system (LABKA), which keeps results for all PSA tests in the region.

### Participant characteristics

Information on age; marital status (married, widower, divorced, and never married); highest achieved education (<10 years [primary and lower secondary school], 10–12 years [upper secondary school or vocational training], >12 years [higher education]); household income adjusted for the number of persons in the household (in tertiles); and working status (employed, unemployed, pensioner, or other) was collected from registries at Statistics Denmark [26,27].

### Practice characteristics

To account for possible differences between practices at baseline, we calculated per practice the number of PSA tests per 1,000 men aged >40 years in the year before the intervention started (2012).

### Sample size

We expected that 75% of the men in the intervention group would receive the genetic prostate cancer risk assessment, and we estimated that 88% of the men would have a normal genetic risk. The sample size was based on an expected reduction of PSA test by 50% in the group with an average genetic risk [17]. This would result in a reduction from 23% to 15% in the overall proportion of men with a PSA test in the follow-up period. This required the inclusion of at least 1,244 men for genetic testing in the intervention arm and, allowing for a design effect of 1.2, provided a study power of 90% with an alpha of 5%.

### Statistical analysis

Data were analyzed using Stata (release 14). For all statistical tests, two-sided hypothesis testing with an alpha of 0.05 was applied. Primary analyses were based on an intention-to-treat principle for both primary and secondary outcomes.

A logistic multilevel model with men nested within practices was used to estimate the odds ratio (OR) as the association between exposure (being in an intervention practice) and outcome. In addition, we performed a time-to-event analysis using a Cox proportional hazards regression model to include the differences in time to the PSA test.

Analyses were adjusted for participant characteristics (age, education, and household income) and for the number of PSA tests per 1,000 men aged >40 years per practice at baseline. Missing values on background characteristics (500 cases, 6.4%) were imputed using a multiple imputation procedure based on the “MICE” approach [28].

Subgroup analyses were performed (1) on men in intervention practices, comparing men with and without a genetic test, and (2) on men with a genetic test, comparing men with a high-risk genetic profile to men with a normal lifetime risk, in relation to outcomes.

### Ethical approval and considerations

The study was conducted according to the Helsinki Declaration principles and was notified to the Danish Data Protection Agency (Journal no. 2011–41–6904). The study has obtained permission from The Danish National Committee on Health Research Ethics (Journal no. 1-10-72-43-12) and was registered at ClinicalTrials.gov (identifier NCT01739062). Only men in the intervention group received printed information about the study from the GP, provided informed consent, and could withdraw from the study at any time and have their blood sample and data destroyed. Patients in the control group were only followed through register information and were not contacted in any way.

## Results

### Study population

**Fig 1:**
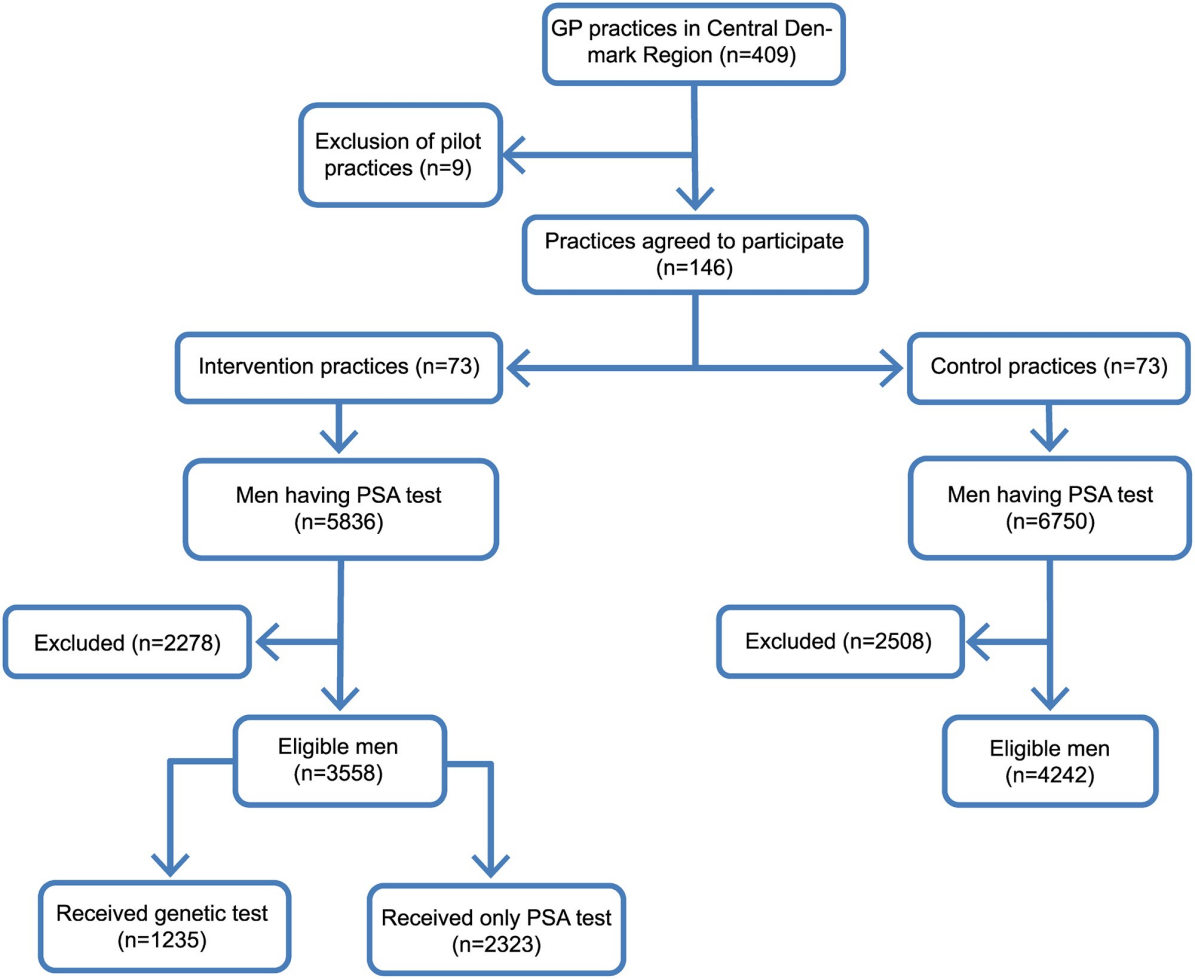
Flowchart. GP, general practitioner; PSA, prostate-specific antigen.

After excluding practices (*n* = 9) that were used for an initial pilot to set up and test the project infrastructure, 146 (36.5% of total) practices in the region accepted the invitation to participate and were randomized (Fig 1).

In the intervention period, 5,836 men in the intervention practices and 6,750 men in the control practices received at least one PSA test (Fig 1). After applying the exclusion criteria, 7,800 eligible men were included in the analyses (intervention, *n* = 3,558; control, *n* = 4,242).

Men who received a PSA test had similar education, income, marital status, and working status between intervention and control practices (Table 1). In intervention practices, fewer men aged <55 (22.9% versus 27.1%) and more men aged >69 years (25.6% versus 21.6%) were eligible for inclusion. Intervention practices had on average slightly more men aged >40 years in their practices than control practices (means 714.4 and 676.0, respectively) and performed fewer PSA tests per 1,000 men per year at baseline (86.5 versus 104.7).

**Table 1:** Description of practice and participant sample at baseline.

Variable	Control (*n* = 4,242)	Intervention			Δ^a^	Total (*n* = 7,800)
		Total intervention (*n* = 3,558)	Received genetic test (*n* = 1,235)	Did not receive genetic test (*n* = 2,323)		
**Cluster level (GP practices)**						
Average number of men age >40 years per practice (SD)	676.0 (412.5)	714.4 (387.1)				695.3 (398.9)
Number of PSA tests per 1,000 men age >40 years at baseline (SD)	104.7 (95.8)	86.5 (67.5)				95.5 (82.9)
Number of practices	73	73				146
**Individual level (men), median years (IQR)**	63.8 (55.1–70.0)	65.1 (57.3–71.0)	62.6 (55.1–68.8)	66.2 (58.3–71.7)		64.5 (56.1–70.5)
Age, years (%)					*	
≤54	27.1	22.9	25.4	21.5		25.2
55–59	13.9	13.3	15.0	12.4		13.6
60–64	17.5	17.1	19.0	16.1		17.3
65–69	19.9	21.1	19.1	22.1		20.4
70≥	21.6	25.6	21.4	27.9		23.5
Highest educational level (%)					*	
<10 years (primary and lower secondary school)	28.2	28.1	23.1	30.9		28.1
10–12 years (upper secondary school or vocational training)	51.8	51.2	53.0	50.2		51.5
>12 years (higher education)	20.0	20.7	23.9	18.9		20.3
Household income (tertiles, %)					*	
Low	33.8	32.8	24.8	37.1		33.4
Medium	33.2	33.5	35.5	32.4		33.4
High	33.0	33.7	39.7	30.5		33.2
Marital status (%)					*	
Married	72.1	74.4	78.9	72.0		73.1
Widower	3.6	3.3	2.5	3.8		3.5
Divorced	10.7	10.5	8.7	11.5		10.6
Never married	13.6	11.8	9.9	12.7		12.8
Working status (%)					*	
Employed	51.5	51.4	58.5	47.6		51.4
Unemployed	2.2	1.7	1.3	1.8		2.0
Pensioner	42.8	44.0	37.6	47.5		43.4
Other	3.5	2.9	2.6	3.1		3.2

^a^Intervention group only: received genetic test versus did not receive test (chi-squared test).

*p < 0.01

Abbreviation: GP, general practitioner; PSA, prostate-specific antigen

### Genetic test

Out of the 3,558 eligible men in intervention practices, 1,235 (34.7%) received a genetic test (Fig 1). This percentage ranged from 0% to 82% between practices (intraclass correlation coefficient [ICC] = 0.37). Men who received the genetic test were younger (median age of 62.6 versus 66.2, *p* < 0.01), more highly educated (23.9% versus 18.9% with higher education, *p* < 0.01), more often married (78.9% versus 72.0%, *p* < 0.01), and employed (58.5% versus 47.6%, *p* < 0.01) and had higher incomes (39.7% versus 30.5% with high income, *p* < 0.01) than men who did not receive the genetic test (Table 1).

Of the men who received a genetic test, 1,047 (84.8%) had a normal (<30% lifetime) risk for prostate cancer, and 136 (11.0%) had a high (≥30% lifetime) risk (Table 2). For 52 men (4.2%), the genetic risk could not be calculated because of missing information about family history of prostate cancer and was included as a separate category as unknown risk.

**Table 2:** Unadjusted primary and secondary outcomes after 2 years’ follow-up.

Repeated PSA test	Control (*n* = 4,242)	Intervention (*n* = 3,558)	*OR*^a^ *(95% CI*, *sign)*	Intervention				
				No genetic test (*n* = 2,323)	Normal genetic risk (*n* = 1,047)	High genetic risk (*n* = 136)	*OR*^b^ *(95% CI)*	*OR*^c^ *(95% CI)*
All men with a PSA test, *N* (%)	1,628 (38.4%)	1,218 (34.2%)	*0*.*95 (95% CI 0*.*78–1*.*14)*	833 (37.5%)	261 (24.9%)	102 (75.0%)	*0*.*65 (95% CI 0*.*5–0*.*78)*	*5*.*96 (95% CI 3*.*96–8*.*97)*
Men with an elevated PSA test, *N* (%)	97 (2.3%)	78 (2.2%)	*1*.*01 (95% CI 0*.*71–1*.*43)*	50 (2.2%)	16 (1.5%)	9 (6.6%)	*0*.*71 (95% CI 0*.*40–1*.*26)*	*3*.*31 (95% CI 1*.*57–6*.*97)*

^a^Control versus intervention.

^b^Average risk versus no genetic test.

^c^High risk versus no genetic test.

Abbreviations: OR, odds ratio; PSA, prostate-specific antigen

### Effect of genetic test on future PSA tests

At 2 years after inclusion, a total of 1,218 men (34.2%) in the intervention practices and 1,628 (38.4%) men in the control practices had a PSA test (OR = 0.95, 95% CI 0.78–1.14, *p* = 0.56) (Table 2). In the intervention group, men with normal genetic risk (24.9%) were less likely to have a repeat PSA test within 2 years than men without a genetic test (37.5%; OR = 0.65, 95% CI 0.54–0.78, *p* < 0.01), whereas men with a high genetic risk were more likely to have a repeat PSA test (75.0%; OR = 5.96, 95% CI 3.96–8.97, *p* < 0.01).

The proportion of all PSA tests (some men had more than one PSA test in the 2-year period) that resulted in an elevated value was similar between the control and intervention group (2.3% versus 2.2%) (OR = 1.01, 95% CI 0.71–1.43, *p* = 0.96). Higher percentages of elevated PSA tests were found in the group of men with high genetic risk (6.6%) than in men without a genetic test (2.2%) (OR = 3.31, 95% CI 1.57–6.97, *p* < 0.01).

In the subgroup of men that had at least one PSA test in the follow-up period, 6.2% of those men had elevated PSA. This was similar between intervention (6.4%, 78/1,218) and control (6.0%, 97/1,628) practices (OR = 1.08, *p* = 0.62). No significant difference was found between men with high genetic risk (8.8%, 9/102) and men with a normal genetic risk (6.1%; 16/261, OR = 1.49, *p* = 0.36).

### Adjusted analysis

After adjusting for confounders, no significant effect was found of the intervention on the number of men with a PSA test at 2 years’ follow-up (OR = 0.95, *p* = 0.53) (Table 3). The number of PSA tests per 1,000 men aged >40 per practice at baseline (OR = 1.02, *p* < 0.01 per 10 extra tests), older age (increasing OR per age group), and a high income (OR = 1.28, *p* < 0.01 compared with the low-income group) were associated with a higher chance of a future PSA test (Table 3).

**Table 3:** Primary and secondary outcomes after 2 years’ follow-up: Adjusted multilevel logistic regression model (OR).

Variable	Men with PSA tests		Men with elevated PSA tests	
	Total population (*n* = 2,846)	Intervention group only (*n* = 1,218)	Total population (*n* = 175)	Intervention group only (*n* = 78)
**Cluster level (practices)**				
Intervention (ref = control)	0.95		1.08	
Number of PSA tests per 1,000 men age >40 years at baseline (×10)	1.02**	1.02**	1.02**	1.03*
**Individual level (men)**				
Genetic test result (ref = no genetic test)				
Normal risk		0.62**		0.64
High risk		8.94**		4.06**
Unknown risk		2.46*		3.41
Age, years (ref ≤54)				
55–59	1.98**	2.11**	3.12**	2.12
60–64	2.59**	2.56**	3.94**	2.60*
65–69	3.41**	3.31**	3.92**	3.01**
70≥	4.01**	4.17**	4.56**	3.04**
Highest educational level (ref <10 years)				
10–12 years (upper secondary school or vocational training)	1.12	1.07	1.19	1.23
>12 years (higher education)	1.10	1.09	1.25	1.63
Household income (ref = low)				
Medium	1.07	1.31**	1.37	2.18*
High	1.28**	1.50**	1.40	1.96

*p < 0.05,

**p < 0.01.

Abbreviations: OR, odds ratio; PSA, prostate-specific antigen; ref, reference group

Within intervention practices, men with a normal genetic risk had a significantly lower chance of having a future PSA test (OR = 0.62, *p* < 0.01), whereas men with a high genetic risk had a significantly higher chance (OR = 8.94, *p* < 0.01) relative to men who did not receive the genetic test (Table 3).

The number of PSA tests with an elevated value followed the same pattern; no significant difference was found between intervention and control practices (OR = 1.08, *p* = 0.65).

## Discussion

### Main findings

The introduction of the genetic risk assessment in general practice did not affect the overall number of men with a normal PSA test receiving a future PSA test within 2 years. Uptake of the genetic test was about one-third, and within this group, more men with a high lifetime prostate cancer risk had a further PSA test compared with men at an average lifetime risk. Men with an average lifetime risk also had fewer PSA tests compared with men who did not receive the genetic test.

### Strengths and weaknesses

Using routinely collected data from registries linked with the personal identifier number ensured complete follow-up and allowed us to use all initially included men in the analyses. The follow-up period of 2 years was not long enough to draw final conclusions about the effect of this intervention on the diagnosis or mortality rate of prostate cancer.

The study was conducted in routine practice and with limited support to the GPs. Consequently, we had no direct control over the criteria for the initial PSA test and whether any digital rectal examinations were performed. Selection bias was evident for the limited proportion of eligible men who received the genetic test, whether by GPs and/or the men consulting, resulting in a relatively young and high-income subsample offered and accepting the test. This may, for example, have been made up of men who were more responsive to the information about lifetime risk. Our study does not provide information about the selection mechanism behind the low uptake. It may be more due to low uptake among some GPs (ICC between practices of 0.37, range of uptake from 0%–82%) rather than participant influences. However, the intention-to-treat analysis minimized this selection bias concerning the primary outcome analysis.

### Comparison with other studies

Until now, a genetic test to assess the risk for prostate cancer has not been tested in a GP setting, and thus no direct comparisons can be made. The Stockholm-1 cohort study tested whether a genetic model based on 36 SNPs could reduce the number of biopsies compared with a nongenetic model based on age, PSA, free-to-total PSA, and family history [15]. Their genetic model reduced the number of biopsies more than the nongenetic model. The proportion of men with an elevated follow-up PSA level in our study population is consistent with downstream effects in previous studies [6,29]. The association that we found between the genetic test result and the number of elevated follow-up PSA tests for those at high risk and reduced tests for those at normal risk illustrates the potential for downstream effect of the intervention. Given the relatively low levels of implementation, however, overall downstream effects were not shown in this study in terms of referrals for prostate biopsy.

Whether to have a PSA test is a complex decision, but studies on decision aids have shown that better-informed decisions can result in fewer men receiving PSA tests [30,31]. We do not know how the GPs used the test results and how they conveyed the result to the men, but other studies suggest that genetic risk assessment increases clinicians’ confidence in managing familial cancer [32]. Recent studies show that both men and GPs still struggle with PSA testing [6,33–36]. In a study among men who had had direct-to-consumer genome-wide profiling to assess prostate cancer risk, Bloss and colleages found that men had a 20% higher intention to take PSA tests with a high lifetime risk [37]. We found that 75% of the men with a high risk actually had a test performed within 2 years, suggesting that men have acted on such knowledge (although the GP may also have influenced such decisions quite significantly), and there is potential for risk stratification to assist routine practice.

### Implications for practice

Implementing the test in routine practice, we did not find an effect on future PSA testing for all men in the intervention practices as a whole. It is important to gain insight into the process of implementation. If uptake continues to be low for either GP or participant reasons, there will be no impact of such risk stratification at the population level, even if there are significant effects in subgroups. However, if barriers to implementation can be removed, further evaluations may show a similar, more appropriate targeting of PSA testing to those at high risk and perhaps an overall reduction of testing. It is important to identify the barriers, address them, and reach a firm conclusion of whether to make a genetic test for lifetime risk of prostate cancer available in general practice.

### Further research

This study showed that it is feasible to integrate a genetic prostate cancer risk assessment in a general practice setting. So far, this has not resulted in an overall reduction in the number of future PSA tests. However, the differences in the numbers of future PSA tests between normal and high lifetime risk groups for those taking up the test suggests that further study is indicated as to whether these effects are replicated, sustained, and large enough to affect an important range of health services research outcomes, such as quality of life, referrals, biopsies, diagnoses of prostate cancer, mortality, impact on overdiagnosis (of indolent pathologies), and resource use effects. Further research is also necessary to explore factors that facilitate or hinder implementation of providing this genetic test to men and how it is used by men (and family members) in deciding on future screening or testing intentions. Based on this, future interventions could be developed that support GPs with the integration of genetic tests in routine practice and men with making informed decisions. It is also unclear whether offering the genetic test had any socioeconomic benefits down the line (e.g., a change in number of transrectal ultrasound (TRUS)-guided biopsies and treatment), which would have to be thoroughly investigated before a full implementation is feasible.

## Conclusion

Offering a genetic test to assess men’s lifetime risk of prostate cancer did not reduce the overall propensity of repeated PSA tests within a 2-year period among men with a normal PSA. However, knowledge of genetic risk reduced the number of PSA tests among those at normal risk and increased the number of PSA tests among those at high risk. Further research is needed to examine how uptake of the test can be supported and whether such an increase can effectively reduce the number of overall future PSA tests and the consequent downstream effects.

## Supporting information

S1 Text CONSORT 2010 Checklist. CONSORT, Consolidated Standards of Reporting Trials.
(PDF)

S2 Text Description of the model for individual estimates of lifetime risk of prostate cancer from data on polygenic susceptibility. (PDF)

## Abbreviations

GP: general practitioner
ICC: intraclass correlation coefficient
IQR: interquartile range
OR: odds ratio
PSA: prostate-specific antigen
ref: reference group
SNP: single nucleotide polymorphism
TRUS: transrectal ultrasound
