# Supplementary material for: The effect of assessing genetic risk of prostate cancer on the use of PSA tests in primary care: A cluster randomized controlled trial
Source: PLoS Med. 2020 Feb 7;17(2):e1003033. doi: 10.1371/journal.pmed.1003033 (PMC7006905; doi:10.1371/journal.pmed.1003033)
Supplement: S2 Text — (PDF) [file pmed.1003033.s002.pdf]

August 8 2017
Michael Væth

APPENDIX

Individual estimates of lifetime risk of prostate cancer from data on polygenic susceptibility

This appendix outlines the calculation of lifetime risk of a disease for an individual at a given age from genetic information on presence or absence of number of risk alleles and population data on mortality and disease incidence. The approach taken here is based on the life-table methods for heterogeneous populations introduced in Vaupel, Manton & Stallard (1979) and further developed in Hougaard (1984). The methods presented can easily be adapted to computation of other absolute risks, e.g. the risk of disease in the next 10 years.

First, the basic notation and a formula for lifetime risk in a homogeneous population are introduced and a general formula for the lifetime risk in a heterogeneous population is then derived using the concept of a frailty. Useful formulas are available if the frailty follows an inverse Gaussian frailty distribution. The polygenic susceptibility model is described next and simulations are used to generate a large sample from the susceptibility distribution predicted by the model. We show that an inverse Gaussian distribution fitted to the frailty distribution derived from the susceptibility model provides an adequate description of the heterogeneity. Finally, implementation of the approach based on the inverse Gaussian distribution is outlined and some individual lifetime risk estimates are presented using recent Danish population data.

Calculation of lifetime risk of a disease

In a homogeneous population let $\bar{\lambda}(t)$ denote the disease incidence rate and let $\mu(t)$ denote the mortality rate. The corresponding integrated rates are denoted $\bar{\Lambda}(t)$ and $M(t)$. This is basically a competing risk situation with two events: disease and death. We assume that the disease has to be diagnosed before death, so the mortality rate represents all causes of death except the disease in question. The following identities are easily established

$$P(\text{alive, disease-free at age } a) = \exp\left(-\int_0^a \left(\bar{\lambda}(s)+\mu(s)\right)ds\right) = \exp\left(-\bar{\Lambda}(a)-M(a)\right) \qquad (1)$$

and

$$\begin{aligned} &\textit{Lifetime risk of disease for an a-years old} \\ &\quad = \int_a^\infty \bar{\lambda}(t)\exp\left(-\int_a^t \left(\bar{\lambda}(s)+\mu(s)\right)ds\right)dt \qquad (2) \\ &\quad = \int_a^\infty \bar{\lambda}(t)\exp\left(-\left\{\bar{\Lambda}(t)-\bar{\Lambda}(a)\right\}-\left\{M(t)-M(a)\right\}\right)dt \end{aligned}$$

If the population is heterogeneous with respect to disease incidence the apparent disease rate, computed as if the population was homogeneous, no longer represents the incidence rate of an individual. The most susceptible individuals in the population become diseased at younger ages and the susceptibility distribution of the healthy population therefore change with age. To describe this effect is convenient to describe the heterogeneity with respect to disease risk by a random variable *Z* such the disease incidence rate of an individual with *Z* = *z* has the form $\lambda(t \mid z) = z\lambda(t)$. The random variable Z is usually denoted the frailty. Individuals with large values of the frailty will have and increased disease incidence. Let $f(z)$ denote the probability density function of *Z*; we assume that $E(Z) = 1$ such that $\lambda(t)$ is the disease rate for an individual with $Z = 1$. We assume moreover that the population is homogeneous with respect to mortality from causes other than the disease. Let $\mu(t)$ denote the common mortality rate (all causes except the disease). For an individual with frailty equal to *z* we have from the result above

$$P(\text{alive, disease-free at age } a \mid Z = z) = \exp\left(-z\Lambda(a) - M(a)\right) \qquad (3)$$

The population average of this probability is obtained by integrated over the distribution of *Z*

$$\begin{aligned} P\left(\text{alive, disease-free at age } a\right) &= \int_z \exp\left(-z\Lambda(a) - M(a)\right) f(z)\,dz \\ &= \exp\left(-M(a)\right) L\left(\Lambda(a)\right) \end{aligned} \qquad (4)$$

where $L(s)$ is the Laplace transform of the density function $f(z)$, i.e.

$$L(s) = \int_0^\infty f(z) \exp(-sz)\,dz,$$

and let $g(s) = \log\left(L(s)\right)$.

A comparison of formula (1) and (4) shows that the apparent disease rate in the population differs from the disease rate of an average individual, a person with *Z* = 1, at birth. This discrepancy reflects the ongoing selection in the population with increasing age. The most susceptible individuals become diseased and the distribution of the frailty in the healthy population therefore changes with age.

From population data we can estimate the average probability of being disease-free and the factor reflecting mortality, $\exp\left(M(a)\right)$, so if the Laplace transform can be inverted the integrated disease rate $\Lambda(t)$ can be recovered from this relation.
The distribution of *Z* among individuals alive and disease-free at age *a* becomes

$$\frac{\exp\left(-z\Lambda(a) - M(a)\right) f(z)}{\exp\left(-M(a)\right) L\left(\Lambda(a)\right)} = \frac{\exp\left(-z\Lambda(a)\right) f(z)}{L\left(\Lambda(a)\right)},$$

and the average frailty among individuals alive, and disease-free at age *a* is obtained as

$$E\left(Z \mid \text{alive, disease-free at age } a\right) = \frac{-L'\left(\Lambda(a)\right)}{L\left(\Lambda(a)\right)} = g'\left(\Lambda(a)\right)$$

Moreover, the lifetime risk of disease for an *a* years old with frailty *z* becomes

$$P\left(\text{ever diseased} \mid \text{alive, disease-free at age } a,\ Z = z\right) =$$
$$\int_a^{\infty} z\lambda(t)\exp\left\{-z\left(\Lambda(t)-\Lambda(a)\right)-\left(M(t)-M(a)\right)\right\}dt$$

In the present application the frailty distribution reflects the genetic variation in susceptibility to prostate cancer. A normal distribution would be an obvious candidate to describe the variation in susceptibility, since the variation mirrors the result of a sum of a large number of small random contributions from different sites. This suggests that a log-normal frailty distribution would work well. Unfortunately, lifetime risk calculations with a log-normal frailty distribution is analytically intractable, so we shall instead consider an inverse Gaussian distribution for which the general approach to lifetime risk calculation outlined above allows easy identification of individual disease rates from population level data.

The inverse Gaussian distribution is a two-parameter distribution on the positive real line. Here we consider a parametrization of the form

$$f\left(z\right) = f\left(z \mid \psi,\theta\right) = \sqrt{\psi\pi^{-1}}\exp\left\{2\sqrt{\psi\theta}\right\}z^{-3/2}\exp\left\{-\theta z - \psi/z\right\}$$

The mean and variance are given by $E\left(Z\right) = \sqrt{\psi/\theta}$ and $Var\left(Z\right) = \left(2\theta\right)^{-1}\sqrt{\psi/\theta}$, see e.g. Johnson et al (1994). A frailty distribution must have mean 1, implying that $\psi = \theta$, and the variance then becomes $\left(2\theta\right)^{-1}$.

When the mean is fixed as 1 the Laplace transform for inverse Gaussian distribution becomes

$$L\left(s\right) = \exp\left\{2\theta\left(1-\sqrt{1+s/\theta}\right)\right\}$$

and

$$g\left(\Lambda(a)\right) = \log\left\{L\left(\Lambda(a)\right)\right\} = 2\theta\left\{1-\sqrt{1+\Lambda(a)/\theta}\right\}.$$

When the frailty distribution is an inverse Gaussian distribution the average probability of being alive and disease-free at age *a* becomes

$$\exp\left\{-M(a) + 2\theta\left(1-\sqrt{1+\Lambda(a)/\theta}\right)\right\}$$

The frailty distribution among individuals alive and disease-free at age *a* is again an inverse Gaussian distribution and the parameters are $\left(\psi,\theta\right) = \left(\theta,\theta+\Lambda(a)\right)$. The average frailty among disease-free at age *a* is therefore

$$\sqrt{\frac{\theta}{\theta + \Lambda(a)}},$$

which is a decreasing function of age.

Formula (1) and (4) both describe the same population level probability and for an inverse Gaussian frailty distribution we therefore have the following identity

$$\exp\left(-\bar{\Lambda}(a)\right) = \exp\left\{2\theta\left(1 - \sqrt{1 + \Lambda(a)/\theta}\right)\right\}$$

This equation can be solved for $\Lambda(a)$ such that the individual rate can be obtained from the population rate:

$$\Lambda(a) = \bar{\Lambda}(a)\left\{1 + \bar{\Lambda}(a)\left(4\theta\right)^{-1}\right\}$$

and consequently

$$\lambda(a) = \bar{\lambda}(a)\left\{1 + \bar{\Lambda}(a)\left(2\theta\right)^{-1}\right\} \tag{5}$$

This relation can also be expressed as

$$\frac{\lambda(a)}{\bar{\lambda}(a)} = 1 + \bar{\Lambda}(a)\,\mathrm{Var}(Z), \tag{6}$$

showing that the disease rate of an average individual (at birth) is always larger than the disease rate seen in at population level. These relationships can be used to obtain lifetime risk estimates for individuals with a given value of *z* from population data.

Distribution of polygenic susceptibility

For prostate cancer a number of common susceptibility variants have been identified through GWAS. Table 1 below shows the 32 SNPs used in the present study. Under suitable assumptions these data can be translated into a frailty distribution.

We assume that the published allele frequencies also apply in Denmark and that there is no interaction between risk alleles both within and between loci. The contributions from each allele to the overall risk can then be added on a log-odds-ratio scale resulting in an aggregated susceptibility distribution, which is back-transformed to a frailty distribution on the original odds-ratio scale and standardized to have mean 1.

Let p_i denote the frequency of the *i*'th risk allele and let x_i denote the logarithm of the corresponding odds ratio. For each variant introduce two random variables y_{i1} and y_{i2} such that $y_{ij} = x_i$ with probability p_i and 0 with probability $1 - p_i$. The total susceptibility of a person then

$$S = \sum_{i,j} y_{ij},$$

and from the assumptions above it follows that

$$E(S) = 2\sum_i x_i p_i \text{ and } Var(S) = 2\sum_i x_i^2 p_i(1-p_i)$$

with an obvious modification if the allele is situated on a sex chromosome.

Internal ID	dbSNP no (VIC/FAM)	Locus/gene	Risk allele	Risk-allele frequency in Europeans	Odds ratio per allele	Reference
SNP-02	rs721048 (A/G)	2p15	A	0.19	1.15	Gudmundsson *et al* (2008)
SNP-03	rs1465618 (C/T)	2p21/THADA	T	0.23	1.08	Eeles *et al* (2009)
SNP-04	rs2660753 (C/T)	3p12	T	0.11	1.18	Eeles *et al* (2008)
SNP-05	rs10934853 (A/C)	3q21.3	A	0.28	1.12	Gudmundsson *et al* (2009)
SNP-06	rs7679673 (A/C)	4q24 /TET2	A	0.55	1.09	Eeles *et al* (2009)
SNP-07	rs17021918 (C/T)	4q22/PDLIM5	C	0.66	1.10	Eeles *et al* (2009)
SNP-08	rs12500426 (A/C)	4q22/PDLIM6	A	0.46	1.08	Eeles *et al* (2009)
SNP-09	rs9364554 (C/T)	6q25	T	0.29	1.17	Eeles *et al* (2008)
SNP-10	rs6465657 (C/T)	7q21	C	0.46	1.12	Eeles *et al* (2008)
SNP-11	rs10486567 (A/G)	7p15 /JAZF1	G	0.77	1.12	Thomas *et al* (2008)
SNP-12	rs2928679 (A/G)	8p21	T	0.42	1.05	Eeles *et al* (2009)
SNP-13	rs1512268 (C/T)	NKX3.1	T	0.45	1.18	Eeles *et al* (2009)
SNP-15A	rs1016343 (C/T)	8q24	T	0.18	1.37	Al Olami *et al* (2009)
SNP-17	rs16902094 (A/G)	8q24	G	0.15	1.21	Gudmundsson *et al* (2009)
SNP-18	rs6983267 (G/T)	8q24	G	0.50	1.26	Yeager *et al* (2007)
SNP-19	rs1447295 (A/C)	8q24	A	0.10	1.62	Amundadottir *et al* (2006)
SNP-20	rs16901979 (A/C)	8q24	A	0.03	2.10	Gudmundsson *et al* (2007a)
SNP-21	rs4962416 (C/T)	10q26 /CTBP2	C	0.27	1.17	Thomas *et al* (2008)
SNP-22	rs10993994 (C/T)	10q11/MSMB	T	0.24	1.25	Eeles *et al* (2008), Thomas *et al* (2008)
SNP-23	rs7127900 (A/G)	11p15	A	0.20	1.22	Eeles *et al* (2009)
SNP-24	rs7931342 (G/T)	11q13	G	0.51	1.16	Eeles *et al* (2008), Thomas *et al* (2008)
SNP-25	rs4430796 (G/A)	17q12 /HNF1B	A	0.49	1.24	Gudmundsson *et al* (2007b)
SNP-26	rs11649743 (A/G)	HNF1B	G	0.80	1.28	Sun *et al* (2008)
SNP-27	rs1859962(G/T)	17q24.3	G	0.46	1.24	Gudmundsson *et al* (2007b)
SNP-28	rs2735839 (A/G)	19q13/KLK2,KLK3	G	0.85	1.20	Eeles *et al* (2008)
SNP-29	rs8102476 (C/T)	19q13.2	C	0.54	1.12	Gudmundsson *et al* (2009)
SNP-33	rs7584330 (A/G)	2q37	G	0.22	1.06	Kote-Jarai et al (2011)
SNP-34	rs6763931 (A/G)	3q23/ZBTB38	A	0.45	1.04	Kote-Jarai et al (2011)
SNP-38	rs130067 (G/T)	6p21/CCHCR1 (G/T)	G	0.21	1.05	Kote-Jarai et al (2011)
SNP-39	rs10875943 (C/T)	12q13/alpha-tubulin,PRPH	C	0.31	1.07	Kote-Jarai et al (2011)
SNP-40 ChrX	rs5919432 (C/T)	Xq12/AR	T	0.81	1.16	Kote-Jarai et al (2011)
SNP-41	rs12543663 (A/C)	8q24	C	0.29	1.28	Al Olami *et al* (2009)

Table 1. The SNPs used in the susceptibility model

Comprehensive data on the distribution of the total susceptibility in the Danish population are not available, but the distribution of *S* is easily derived by a computer simulation. The frailty distribution is then obtained by applying an exponential transformation and then scaling the result such that the mean value is 1, i.e. $Z = \exp(S) / E\{\exp(S)\}$. Figure 1 shows the distribution of Z based on 500,000 simulations together the best fitting inverse Gaussian distribution with mean 1. The best-fitting inverse Gaussian distribution was found by maximum likelihood estimation and had $\hat{\theta} = 1.324$. Minor systematic deviations can be identified in Figure 1, but overall the inverse Gaussian distribution seems to provide a very good approximation to the distribution derived from the simulations.

Implementation of the methodology

In the formulas above, the age is a continuous variable, but population data on mortality and disease incidence are usually only available with age categorized in 1-year or 5-years

intervals, so the calculations have to be adapted to categorical population data. In this section the basic steps of this implementation are described when age is categorized in 1-year intervals. Obvious modifications are needed, if 5-years age intervals are used.

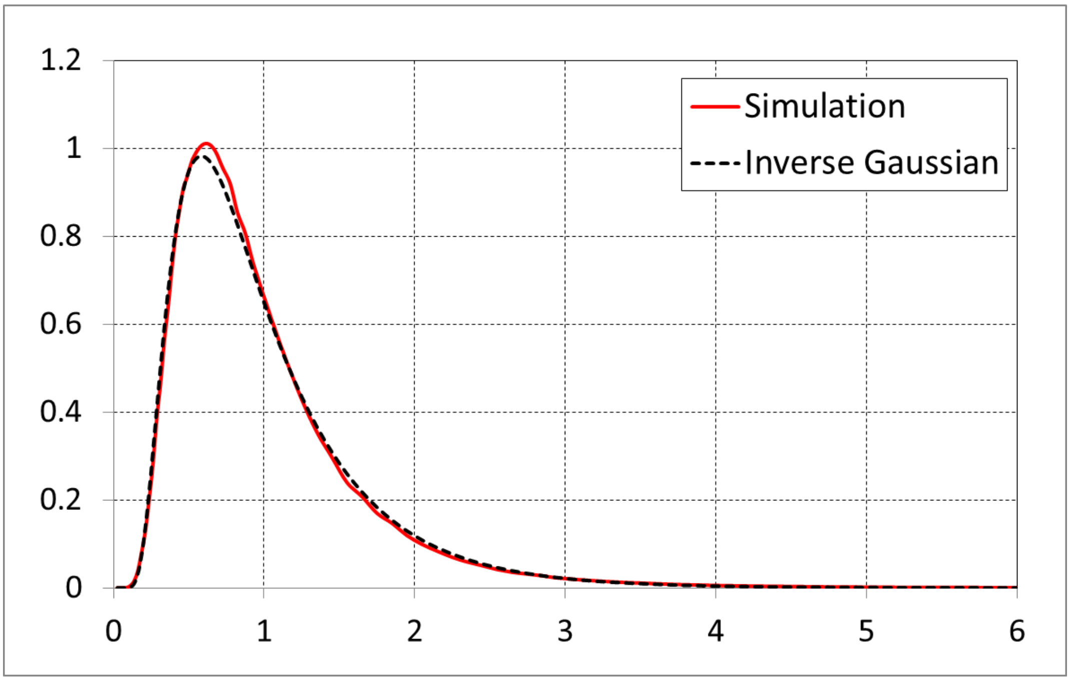

Figure 1. The frailty distribution derived from the 500,000 simulations of the susceptibility model. Superimposed is the probability density function of the best fitting inverse Gaussian distribution with mean 1.

Population data on mortality is often summarized by a life table that describes how a hypothetical cohort of 100,000 newborns is reduced by mortality. The number of surviving individuals in this cohort at age *x* is usually denoted l_x with $l_0 = 100000$. Published Danish life tables gives l_x for $x = 0,1,2,\ldots,99$ (StatBank Denmark 2017, Table HISB8).

The probability of surviving until age *x* is $S_T(x) = l_x / l_0$, where the subscript *T* indicates that survival is from all causes of mortality, i.e. *T*otal mortality. The integrated total mortality rate is obtained as $M_T(x) = \log(-S_T(x))$, and the mortality rate at age *x* becomes

$$m_T(x) = M_T(x+1) - M_T(x)$$

Vital statistics also include information on cause of death (StatBank Denmark 2017, Table DOD1). Let $\pi_P(x)$ denote the proportion of all deaths at age *x* with prostate cancer as cause of death, then

$$\pi_P(x) = \frac{d_P(x)}{d_T(x)},$$

Where $d_P(x)$ and $d_T(x)$ denote the number of death from prostate cancer and the total number of death at age *x*. The mortality rate from all causes except prostate cancer is then $m_{\overline{P}}(x) = m_T(x)\left(1-\pi_P(x)\right)$, and let $M_{\overline{P}}(x)$ denote the corresponding integrated mortality rate

$$M_{\overline{P}}(x) = \sum_{y=0}^{x-1} m_{\overline{P}}(y)$$

For a number of cancers, including prostate cancer, the cancer registry each year publishes the number of cases and the incidence rate by age and sex (Cancerregisteret 2017). Let $\overline{\lambda}_P(x)$ denote the population prostate cancer incidence rate at age x and let $\overline{\Lambda}_P(x)$ denote the corresponding integrated rate

$$\overline{\Lambda}_P(x) = \sum_{y=0}^{x-1} \overline{\lambda}_P(y)$$

The prostate cancer incidence rate for an individual with $Z=1$ is then obtained from (5) as

$$\lambda_P(x) = \overline{\lambda}_P(x)\left\{1+\left(2\cdot 1.324\right)^{-1}\left(\overline{\Lambda}_P(x)+\overline{\Lambda}_P(x+1)\right)/2\right\},$$

where the average value of the integrated rate has been used to account for the fact that the rate $\lambda_P(x)$ applies to the age interval from x to $x+1$. The prostate cancer rate for an individual with frailty *z* is then $\lambda_P(x \mid z) = z\lambda_P(x)$. We now consider the composite event *diagnosis of prostate cancer or death from all causes except prostate cancer.* The event rate at age *x* for an individual with frailty *z* becomes

$$m_E(x \mid z) = z\lambda_P(x) + m_{\overline{P}}(x) \tag{9}$$

The probability of a composite event before age $x+1$ given that the individual is alive and event free at the *x* year birthday is then obtained as

$$q_E(x \mid z) = 1 - \exp\left(-m_E(x \mid z)\right). \tag{10}$$

Moreover, the probability that a composite event is a prostate cancer diagnosis given that the composite event is experienced by an individual with frailty *z* at age *x* is estimated by

$$\rho_P(x \mid z) = \frac{z\lambda_P(x)}{m_E(x \mid z)} \tag{11}$$

Finally, compute the unconditional probability of event-free survival until age *x* by recursively using

$$S_E(x+1 \mid z) = S_E(x \mid z)\{1 - q_E(x \mid z)\} \tag{12}$$

starting with $S_E(0) = 1$.

The lifetime risk of prostate cancer for an individual with frailty *z* and event-free at age *a* can now be obtained as

$$LR(a,z) = \sum_{x=a}^{\omega} S_E(x \mid z) q_E(x \mid z) \rho_P(x \mid z) \Big/ S_E(a \mid z) \tag{13}$$

The risk of prostate cancer before age *t* for an individual with frailty *z* who is alive and disease-free at age *a* is obtained by restricting the summation to $x \le t$.

Figure 2 shows the lifetime risk of prostate cancer as a function of age *a* at which the individual is alive and disease free. Predictions for three individuals with frailty 0.29, 1, and 2.67, respectively, are shown. The values 0.29 and 2.67 correspond approximately to the lower and upper 2.5 percentile of the frailty distribution at birth. The results in Figure 2 are based on Danish population data for the years 2008-9 (StatBank Denmark 2017, Cancerregisteret 2017). The lifetime calculations used 5-years intervals, the last interval included the ages from 95 and above. The calculations have been implemented in a spreadsheet.

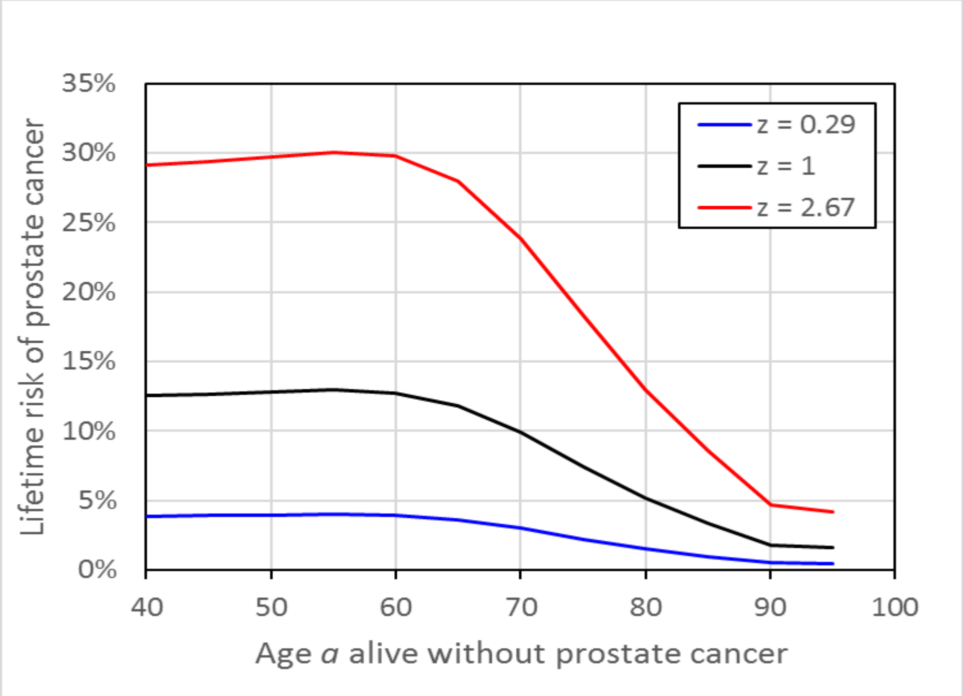

Figure 2. Lifetime risk of prostate cancer as a function of age for individuals with frailty 0.29, 1, and 2.67.

References

Cancerregisteret. http://esundhed.dk/sundhedsregistre/CAR/Sider/Cancerregisteret.aspx (accessed August 8 2017)

Hougaard P (1984). Life table methods for heterogeneous populations: Distributions describing heterogeneity. *Biometrika* **71**, 75-83.

Johnson NL, Kotz S & Balakrishnan (1994). Continuous univariate distribution, Volume 1. Second edition. Chapter 15. J Wiley & Sons, Inc New Yok.

StatBank Denmark: http://www.statistikbanken.dk/ (accessed August 8 2017)

Vaupel JW, Manton KG & Stallard E (1979). The impact of heterogeneity in individual frailty on the dynamics of mortality. *Demography* **16**, 439-456.
